# Long read genome assembly of *Automeris io* (*Lepidoptera: Saturniidae*) an emerging model for the evolution of deimatic displays

**DOI:** 10.1093/g3journal/jkad292

**Published:** 2024-02-07

**Authors:** Chelsea Skojec, R Keating Godfrey, Akito Y Kawahara

**Affiliations:** McGuire Center for Lepidoptera and Biodiversity, Florida Museum of Natural History, University of Florida, Gainesville, FL 32611, USA; Department of Biology, University of Florida, 220 Bartram Hall, Gainesville, FL 32611, USA; McGuire Center for Lepidoptera and Biodiversity, Florida Museum of Natural History, University of Florida, Gainesville, FL 32611, USA; McGuire Center for Lepidoptera and Biodiversity, Florida Museum of Natural History, University of Florida, Gainesville, FL 32611, USA; Department of Biology, University of Florida, 220 Bartram Hall, Gainesville, FL 32611, USA

**Keywords:** eyespots, antipredator, Bombycoidea, behavior, deimatism, Io moth

## Abstract

*Automeris* moths are a morphologically diverse group with 145 described species that have a geographic range that spans from the New World temperate zone to the Neotropics. Many *Automeris* have elaborate hindwing eyespots that are thought to deter or disrupt the attack of potential predators, allowing the moth time to escape. The Io moth (*Automeris io*), known for its striking eyespots, is a well-studied species within the genus and is an emerging model system to study the evolution of deimatism. Existing research on the eyespot pattern development will be augmented by genomic resources that allow experimental manipulation of this emerging model. Here, we present a high-quality, PacBio HiFi genome assembly for Io moth to aid existing research on the molecular development of eyespots and future research on other deimatic traits. This 490 Mb assembly is highly contiguous (N50 = 15.78 mbs) and complete (benchmarking universal single-copy orthologs = 98.4%). Additionally, we were able to recover orthologs of genes previously identified as being involved in wing pattern formation and movement.

## Introduction

*Automeris* moths are a charismatic genus belonging to the giant silk moth family (Saturniidae), characterized by large, striking eyespots on their hindwings. The Io moth (*Automeris io*) is a well-studied, North American species that is polyphagous as larvae, feeding on a variety of host plants (Bernays and Janzen 1988). Adult Io moths do not feed and, like most silk moths, have a brief life span no greater than a few weeks, during which time they focus on reproduction and oviposition. Females lay their eggs in clusters on leaves, and once hatched, caterpillars consume large amounts of leaves as they develop. Io moth caterpillars are known for their bright green coloration and urticating spines, which cause human skin swelling, itching, and burning (Diaz 2005).

Io moths are cryptically colored with forewings that resemble dead leaves. They show sexual dimorphism in forewing coloration, with females being generally dark brown in color and males often bright yellow. Hindwings of *Automeris* are often brightly colored and have conspicuously colored eyespots used to startle predators (Manley 1990). The sudden reveal of bright colors or patterns is considered a unique antipredatory behavior, a deimatic defense. Deimatism is distinguished from aposematism in that the traits function to confuse or startle a potential predator, causing a momentary distraction allowing prey to escape. The combined physical traits and behavior are thought to trigger unlearned avoidance in the predator (Umbers and Mappes 2016; Drinkwater *et al*. 2022). The Io moth is an emerging model organism for research into deimatic displays. Two primary components make up their display: sudden movement upon subjugation and the revealing of conspicuous eyespots. Because thoracic muscles controlling wing movement are part of the display, it is possible that the motion component was co-opted from motor function related to flight. Previous research suggests that, given the complexity of the 2 components involved in deimatic displays, motion and conspicuous coloration evolved separately (Holmes *et al*. 2018).

Given the broad interest in eyespot color and pattern development, a genome of the Io moth will serve as a framework for future research on genes involved in patterns, shapes, and colors. To ensure that the genome is sufficient quality for these kinds of analyses, we performed a draft annotation and searched for orthologs of genes that could be related to deimatism, focusing on those characterized as being involved in wing coloration and patterning, and muscle movement (Monteiro *et al*. 2013; Cao *et al*. 2019; Murugusen *et al*. 2022). The genome reported here will allow future studies to link the display components of interest to underlying genes. As more genomes become available, a comparison of this genome with other deimatic taxa and nondeimatic taxa can be used to study the evolution of gene functions as they relate to antipredatory phenotypes.

## Materials and methods

### DNA isolation and sequencing

An adult male Io moth was collected in Gainesville, Florida, and vouchered at the Florida Museum of Natural History's McGuire Center for Lepidoptera and Biodiversity (LEP-86049). The specimen was stored at −80 °C until DNA was isolated from thoracic tissue using the Qiagen DNeasy Blood and Tissue Kit (Cat. # 69504; see Supplementary Material online for details). The University of Florida Interdisciplinary Center for Biotechnology Research (ICBR; RRID:SCR_019152) performed SMRT bell library preparation and sequenced the material using PacBio SEQUEL IIe.

### Genome size

Genome size and heterozygozity were estimated from PacBio consensus reads using K-mer counter v.3.2.1 (RRID: SCR_001245; Vurture *et al*. 2017). A k-mer length of 23 (-m 23) was used to create a histogram of k-mer frequencies and visualized using GenomeScope 2.0 (RRID:SCR_017014).

### Assembly

PacBio consensus reads were assembled using the de novo assembler, HiFiasm v.0.16.1 r307 (RRID:SCR_021069; Cheng *et al*. 2021). Assembly contiguity was assessed using the assembly_stat.py script (Manchanda *et al*. 2020) and completeness was assessed using benchmarking universal single-copy orthologs (BUSCO v.5.2.0) with 5,286 putative single-copy genes from the lepidoptera_odb10.2019-11-20 database (RRID:SCR_015008; Simão et al. 2015; Manni *et al*. 2021). Despite using the most aggressive duplicate purging setting in Hifiasm (option -l 3), BUSCO detected duplicated orthologs at a rate of 6% in this primary assembly. Therefore, we attempted to further collapse allelic variation using the Purge Haplotigs pipeline (purge_haplotigs v.1.1.2; Roach *et al*. 2018). We first generated a coverage histogram to choose a minimum, median, and maximum read depth cutoff value for purging by mapping raw reads to the primary assembly using minimap v.2.21 (RRID:SCR_018550; Li 2018). Contigs were assigned as haplotigs if 80% of the contig showed diploid-level coverage (-s 80) and discarded if coverage was 80% above or below the read depth cutoffs (-j 80). This purging step was performed twice to create the final assembly.

Potential contamination in the assembly was assessed using BlobTools v1.0 (RRID:SCR_017618; Laetsch and Blaxter 2017). Genome assembly contiguity was assessed by performing syntenty analysis using MUMmer (RRID:SCR_018171; Marçais *et al*. 2018) to align Io moth contigs with the chromosome-level assembly of the small emperor moth (*Saturnia pavonia*). The small emperor moth has 31 chromosomes, which is 3 more than what is reported for the Io moth (*N* = 29, Cook 1910), but it is the closest nondomesticated relative for which a chromosome-level assembly is available. We limited the minimum alignment length displayed in the synteny plot to 300 bp using the delta-filter utility in MUMer (-l 300) and plotted synteny using a custom R script available from Qin (2020).

### Annotation

We ran RepeatModeler 2.0.4 (RRID:SCR_015027; Flynn *et al*. 2020) with structure-based LTR discovery (-LTRStruct) to identify repeated elements. RepeatMasker (RRID:SCR_012954) was then used to mask repeat regions of the assembly, creating a soft-masked genome to be used for all downstream analyses. The BRAKER2 pipeline (v2.1.5; RRID:SCR_018964; Hoff *et al*. 2019) with protein sequences from the NCBI *Bombyx mori* Annotation Release 101 was used for structural annotation. .This pipeline relies on BamTools (RRID:SCR_015987; Barnett *et al*. 2011), GeneMark-EP+ (RRID:SCR_011930; Bruna *et al*. 2020), DIAMOND (RRID:SCR_016071; Buchfink *et al*. 2015), and Augustus (RRID:SCR_008417; Stanke *et al*. 2008). Annotation statistics were summarized using gFACs (RRID:SCR_022017; Caballero and Wegrzyn, 2019). BUSCO v.5.2.0 was used to assess the completeness of this annotation as described for the genome assembly.

### Genes related to deimatism

**Fig. 1.**
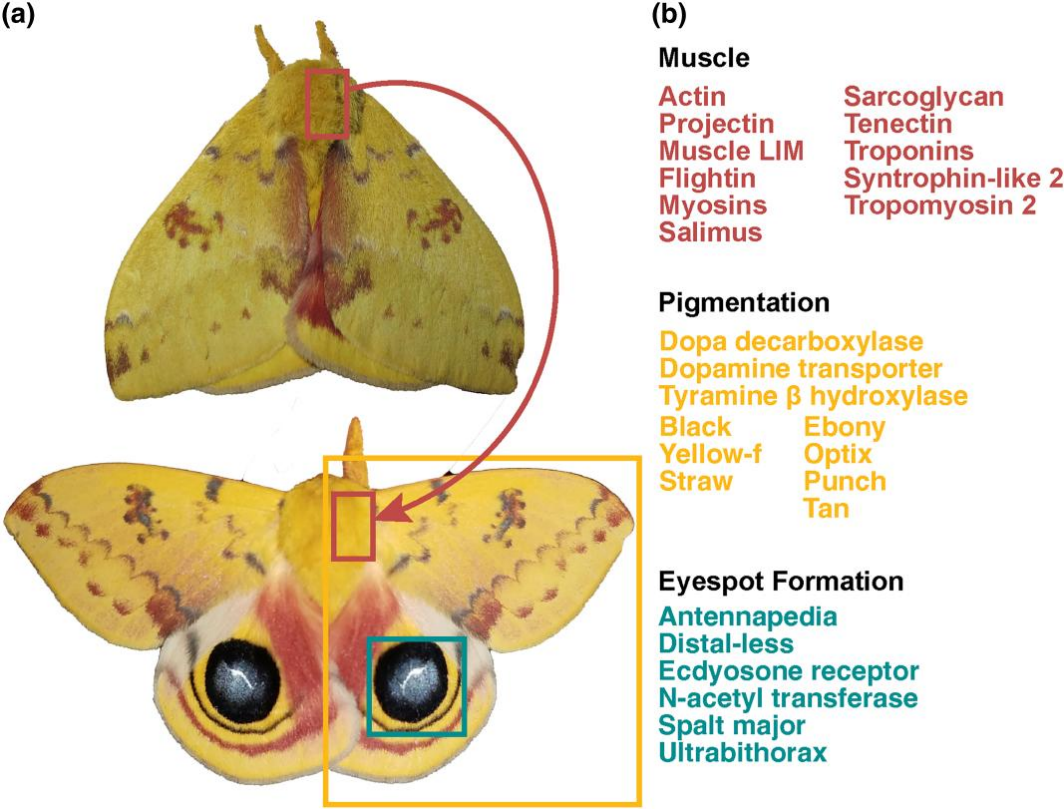
a) The Io moth (*Automeris io*) in resting cryptic state with hindwings concealing eyespots (top) and deimatic display with wings up revealing eyespots (bottom). b) Genes of interest for muscle movement, pigmentation, and eyespot formation. Resting cryptic state image in a) by Jacy Lucier, own work, CC BY-SA 4.0, https://commons.wikimedia.org/w/index.php?curid=85979057. Open wing display image in a) by lead author Chelsea Skojec.

The Io moth is well-known for its deimatic defense behaviors, and therefore, we examined whether genes associated with wing pattern and muscle movement could be recovered from our assembly. We focused on a subset of highly conserved genes associated with melanization pathways (Sugumaran and Barek 2016), eyespot development (Monteiro *et al*. 2013; Murugusen *et al*. 2022), and structural constituents of the muscle (Gene ontology term GO:0008307, Ashburner *et al*. 2000; The Gene Ontology Consortium 2023; Fig. 1, Supplementary Tables 1 and 2). We expected to recover these genes from the draft annotation and identify them as orthologs of those functionally characterized in *Drosophila melanogaster* and of annotated genes from more closely related moth species. To do this, we used OrthoFinder, which infers orthogroups and builds ortholog gene trees from a set of peptide files for species of interest (Emms and Kelly 2015). We included 2 related moth species for which NCBI RefSeq annotations are available, the domestic silkworm (*B. mori*) and the tobacco hornworm (*Manduca sexta*), and 2 outgroups from different insect orders, the cardinal beetle (Coleoptera: *Pyrochroa serraticornis*) and the fruit fly (Diptera: *D. melanogaster*). Annotated peptide FASTA files from 7 additional Lepidoptera available from the Darwin Tree of Life project (Supplementary Table 1; Darwin Tree of Life Project Consortium 2022) were downloaded from the Ensembl Genome Browser. To ensure that comparable annotations were being used to determine orthogroups, we chose species for which annotations were predicted using a BRAKER2 pipeline. FASTA files were filtered to retain only the longest sequence of each peptide before being analyzed by OrthoFinder. Because many of these genes have been functionally characterized in *D. melanogaster*, Fly Base gene IDs (FlyBase Consortium 2003) were used to identify orthogroups containing genes of interest.

## Results and discussion

### Sequencing and genome assembly (assembly stats and BUSCO)

Sequencing resulted in over 1.9 million HiFi reads, with the majority of reads being 5–15 kb with a mean read length of 7.2 kb. Our primary assembly was 500 Mb with an N50 of 15.78 Mb (Table 1). Removing putative haplotigs from the draft assembly resulted in a 1.96% reduction in overall size (490 Mb; Table 1) but improved the recovery of single-copy orthologs, reducing detected duplicated BUSCOs from 6% in the primary assembly to 4.7%. The N50 of 15.78 kb and GC content of 36.3% are comparable with other Saturniidae assemblies (Table 1). Repeat modeler identified 50.36% of the assembly as repeated elements (Supplementary Table 3). This is higher than some previously reported lepidoptera genomes (Kawamoto *et al*. 2019; Singh *et al*. 2022; Hundsdoerfer *et al*. 2023), but this may be due in part to the uneven taxonomic representation in repeated element reference databases (Sproul *et al*. 2022) . Recent research has demonstrated that, in insects, specifically 25–85% of repeated elements were unclassified (Sproul *et al*. 2022). A contamination check with BlobTools showed that taxonomically identifiable sequences matched arthropods and not plants or fungi, indicating an uncontaminated assembly (Supplementary Fig. 2).

**Table 1.** Assembly statistics for *Automeris io* and 2 related saturniid moths.

	*Automeris io*		*Bombyx mori*	*Saturnia povia*
Assembly name	Curated	First draft	Bmori_2016v1.0	ilSatPavo1.1
Total sequence length (bps)	490,212,539	500,025,378	460,349,660	489,898,868
N50 (Mbs)	15.78	15.78	16.8	17.68
Contigs	204	602	697	72
GC content	36.3	36.35	36.3	35.8

**Fig. 2.**
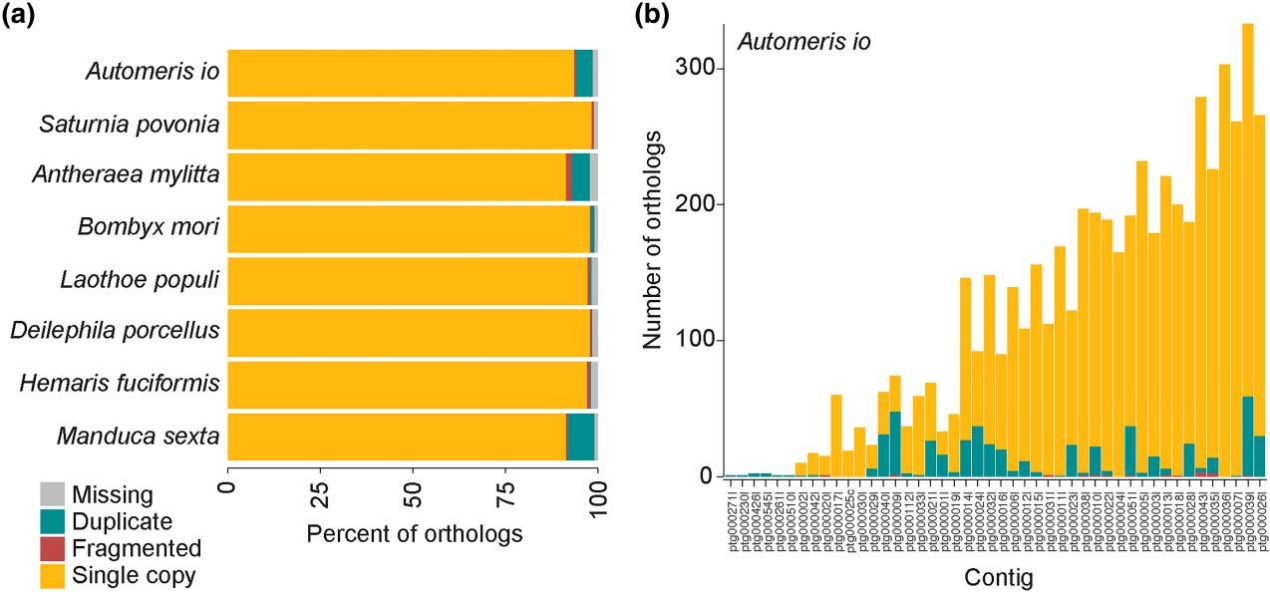
Genome assembly completeness assessed by BUSCO with the OrthoDB v10 Lepidoptera database. a) Assembly completeness comparison with other bombycoid moths. b) Distribution of BUSCO genes colored by classification type (single copy, duplicate, fragmented, or missing) on contigs of the Io moth–curated assembly (ranked from smallest to largest).

A BUSCO analysis showed 98.4% genome completeness with 93.7% single-copy, 4.7% duplicated, and 1.7% missing BUSCOs (Fig. 2a). This duplication percent is higher than some, but not all, de novo Lepidoptera assemblies and may be due to heterozygosity that was not collapsed in the final assembly. The kmer plot coverage revealed a somewhat high heterozygosity of 1.72%, which might be a result of using a wild-caught Io moth (Supplementary Fig. 1). Additionally, the mean read length of 7.2 kb is relatively short for PacBio HiFi sequencing. These variables might make it difficult to collapse variation across chromosomes into a single assembly. Therefore, we looked at the distribution of duplicated BUSCO hits across the assembly contigs and found that a number of them mapped to smaller contigs without any single-copy ortholog hits (Fig. 2b). A synteny analysis revealed high contiguity between the Io moth assembly contigs and the small emperor moth chromosomes (Supplementary Fig. 3) but also indicated that there could be areas of heterozygosity that remain uncollapsed in our final assembly. Synteny with a chromosomal-level assembly from a closer relative will be necessary to confidently collapse these areas, and therefore, we retained them in our assembly so as not to remove potentially useful information.

### Annotation

The structural annotation based on all *B. mori* RefSeq proteins recovered 17,622 protein-coding genes with a 96.1% BUSCO score. However, this resulted in a duplicated BUSCO percent that was considerably higher (11.0%) than that of other moth genomes annotated with the BRAKER2 pipeline (∼1.0–3.8%; Supplementary Table 1). We, therefore, reannotated the genome using only the longest of each of the *B. mori* RefSeq transcripts, which recovered 17,560 predicted protein-coding genes, nearly one quarter of which are monoexonic (Table 2). This produced a BUSCO completeness score of 95.6% (a difference of 23 genes from the previous annotation), while reducing predicted, duplicate BUSCOs to 5.9%. This duplication rate is still higher than that of some other BRAKER2-based annotations but lower than that of the well-curated *M. sexta* RefSeq annotation (Supplementary Table 1). Notably, this draft annotation showed a relatively low number of missing BUSCOs and was sufficient for downstream analyses looking at assembly quality, but gene expression data from different life stages will be necessary to perform a high-quality annotation required for comparative work.

**Table 2.** Summary statistics for genes annotated using BRAKER2 analyzed with gFACs.

Number of genes	17,560
Monoexonic	3,386
Multiexonic	14,174
Positive strand genes	8,752
Monoexonic	1,609
Multiexonic	7,143
Negative strand genes	8,808
Monoexonic	1,777
Multiexonic	7,031
Gene sizes (bp)	
Mean	8,286.179
Median	4,861
Mean exon	232.588
Median exon	158
Mean monoexonic	916.247
Median monoexonic	660
Mean multiexonic	10,046.768
Median multiexonic	6,839

### Deimatism genes of interest

**Fig. 3.**
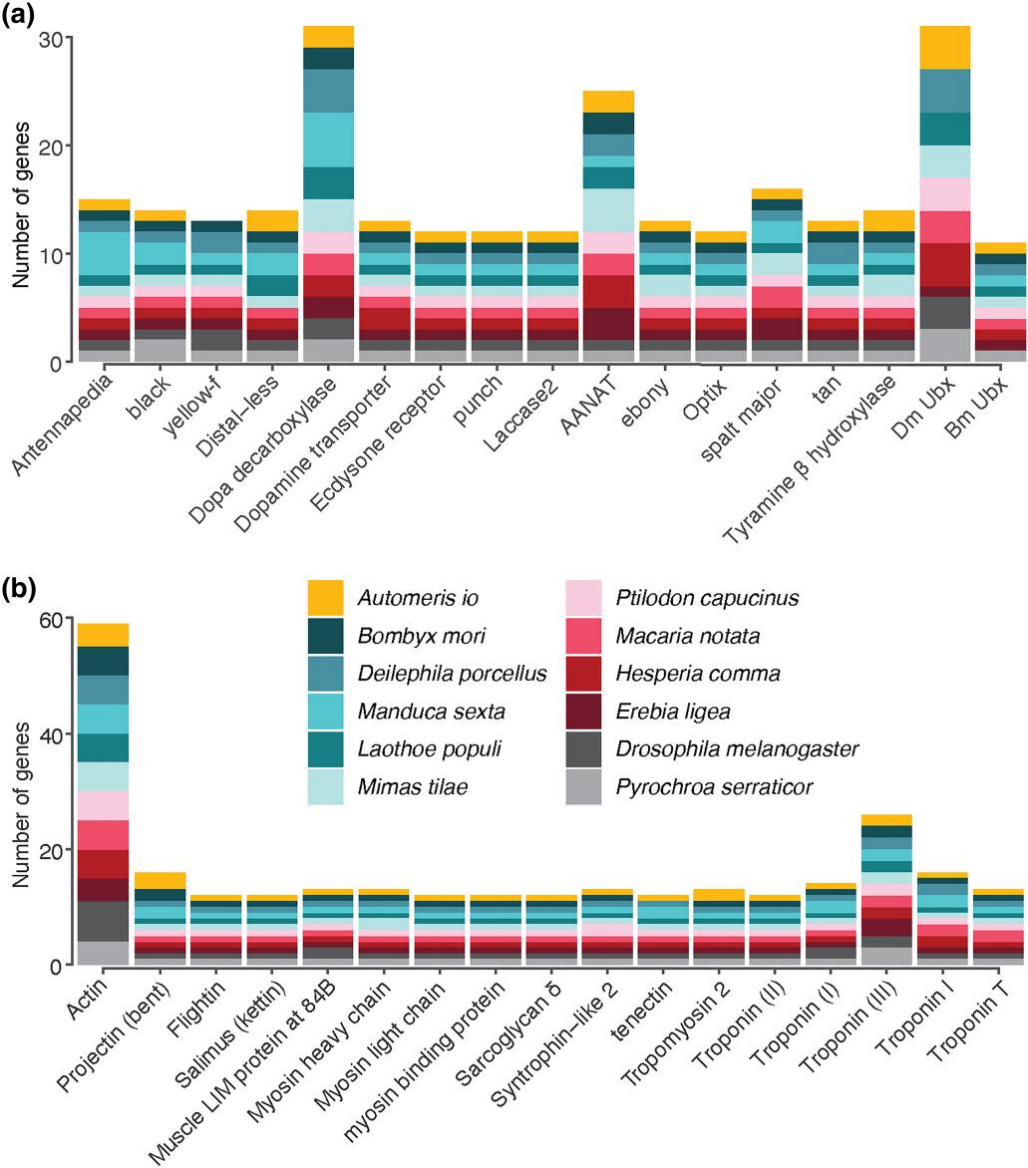
Orthogroups for a subset of genes of interest to deimatism. a) Genes of interest to wing pigmentation and eyespot development. b) Genes of interest as structural components of the muscle important for wing movement.

In performing a draft annotation and searching for genes of interest to deimatism, our aim was to ensure that we could recover genes that may be involved in deimatic displays from our assembly, demonstrating the quality of this genome for comparative research. Orthologs for all focal genes of interest to eyespot development were recovered from our Io moth annotation (Fig. 3). This is reassuring, as most of these genes perform functions beyond pigmentation or patterning, and remain conserved across Lepidoptera through purifying selection (Kuwalekar *et al*. 2020). For example, phenotypic differences in melanization patterns are often generated through the regulation of core melanin synthesis genes rather than through sequence divergence (Yoda *et al*. 2014; Abolins-Abols *et al*. 2018). While gene duplication events may underlie trait evolution, the recovery of multiple copies of particular genes from our draft annotation could also indicate uncollapsed regions of the assembly. We, therefore, took a closer look at 2 genes, distal-less and tropomysin2, for which related species contain only one copy, but 2 were recovered as orthologs from our draft annotation.

Orthologs for all but one focal gene of interest for pigmentation were recovered from the annotation. Interestingly, we did not recover sequences that fell within the orthogroup containing the *D. melanogaster* genes *yellow-f* and *yellow-f2*, and the *B. mori* gene *yellow-fa* (Fig. 3a). We chose to look at the *yellow-f*-like orthogroup because of a documented role in melanization (Han *et al*. 2002; Wittkopp *et al*. 2002) and wing patterns (Ferguson *et al*. 2011). However, the *yellow* gene family is large and ancient and, where characterized, protein products appear to play a variety of roles in development and behavior (Ferguson *et al*. 2011). From our annotation, we did recover 4 putative orthologs to members of the yellow gene family containing *D. melanogaster yellow-h* and *B. mori yellow-12*. The large number of high-quality genome assemblies available for the major Lepidopteran clades (Darwin Tree of Life Project Consortium 2022) suggests that an updated assessment of yellow gene family evolution in moths is possible.

**Fig. 4.**
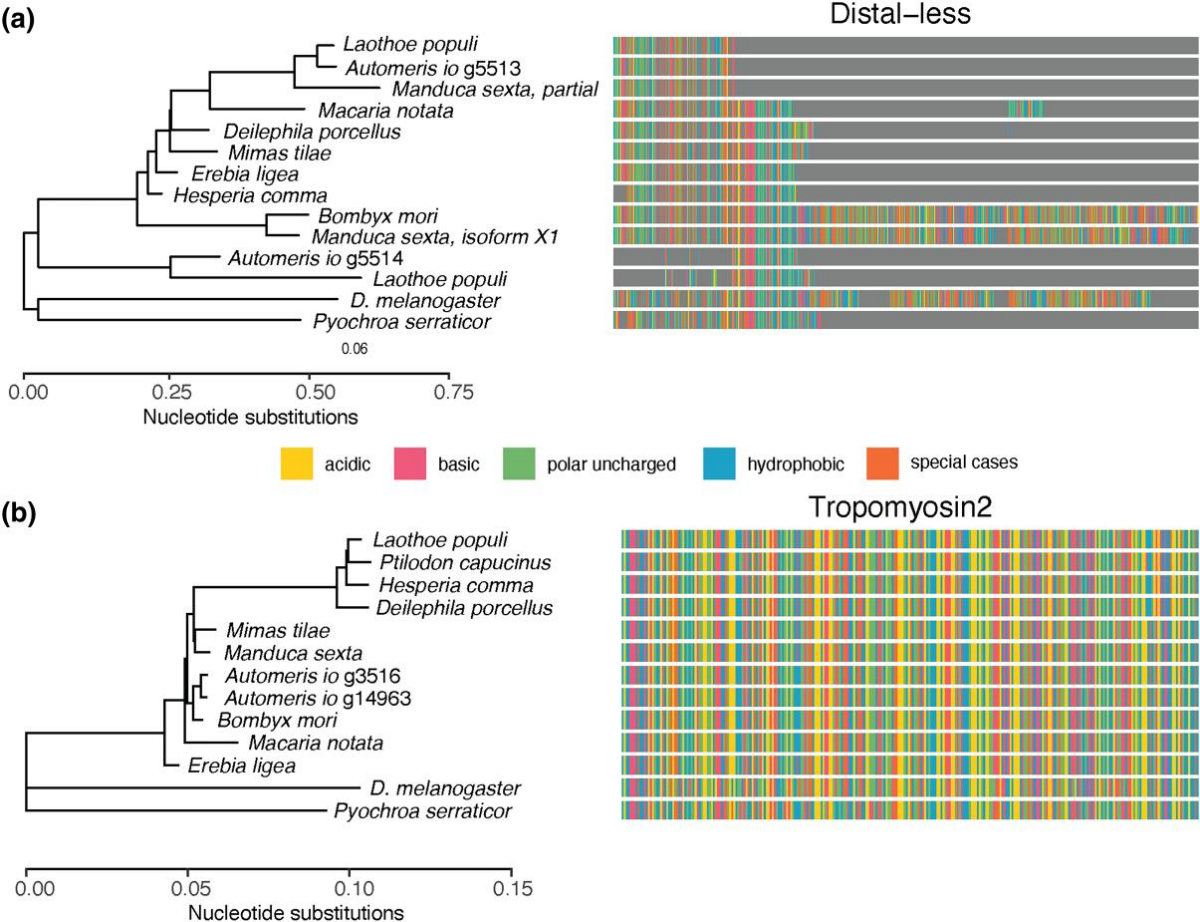
OrthoFinder gene trees for 2 of the candidate genes of interest to deimatism. a) The distal-less gene tree showing that 2 copies recovered from the Io moth annotation are likely to be a contiguous sequence containing a stop codon. b) Two tropomyosin2 genes identified from the Io moth are identical and most similar in sequence to the domestic silkmoth. Io moth branch names appended with Braker2 gene IDs for reference.

OrthoFinder suggested that there were 2 distal-less (*Dll*) genes in the Io moth, but it appears that the draft annotation identified the gene as 2 because of a stop codon. The *M. sexta* annotation also includes a partial *Dll* gene of the same length, but a longer isoform more closely related to that of *B. mori* is included in the *M. sexta* annotation (Fig. 4a). While stop codon readthrough is reportedly common from a number of fly species (e.g. Dunn *et al*. 2013; Jungries *et al.* 2016), we are not aware of its frequency being documented in Lepidoptera. More generally, there appears to be variation in the recovery of the *Dll* gene across the Lepidoptera annotations used in this study (Fig. 2a). Thus, while we could recover this gene from our assembly, it illustrates the necessity of using gene expression data from different tissues and life stages to identify potential isoforms of genes for comparative analysis.

Interest in the morphology, development, and function of thoracic muscle has focused primarily on insect flight (e.g. Dickinson and Tu 1997; Dickerson *et al*. 2014; Gong *et al*. 2020). Interestingly, the structure of the saturniid flight muscle suggests an adaptation for powerful instead of fast movement (Carnevali and Reger 1982). The Io moth display is characterized by a fast lifting of the forewing (Blest 1957), and it is possible that selection has acted on some aspect of muscle movement in the evolution of this deimatic defense. Structural aspects of the muscle are but one phenotypic variable involved in the reveal display, and any or all components of the sensory-motor behavior may have faced selective pressure. But because genes controlling the structural components of the muscle are relatively well-characterized, we focused on these. Indeed, all orthologs for the focal genes of interest were recovered from our *Automeris io* annotation (Fig. 3b).

OrthoFinder detected 2 copies of the genes encoding tropomyosin2 (*Tm2*), which is a single-copy gene in related moths (Fig. 3b; Supplementary Table 2). The copies recovered from the Io annotation are identical and show the closest similarity with the *B. mori Tm2* (Fig. 4b). These 2 copies of *Tm2* are annotated from different contigs in the assembly; both of these contigs in the top 25 are largest in length and contain many single-copy BUSCOs (ptg000012l and ptg000032l in Fig. 2b). Also, while the Purge Haplotigs analysis suggests that ptg000032l is a top match for ptg000012l, their alignment score is only 21.44. Last, they do not map to the same chromosome of the *S. pavonia* genome (Supplementary Fig. 3). This suggests that while we can confidently recover the tropomyosin2 gene from our assembly, it may not occur as a single copy.

### Conclusion

Our high-quality genome assembly of *Automeris io* provides a basis needed to address diverse sets of biological questions. This genome assembly will serve as a valuable tool for future studies investigating the genomic basis of eyespots in *Automeris* and for comparison with other genera. Furthermore, genes involved in fast muscle movement identified in this genome may serve as a basis for subsequent investigations into the evolution of deimatic displays. The increasing availability of a high-quality, annotated genome allows for comparative analyses of genes and gene families across insect taxa, and future research will incorporate additional genomes to this set along with gene expression analysis to further test the evolution of traits involved in deimatism in *Automeris* moths.

## Supplementary Material

jkad292_Supplementary_Data

## Data Availability

PacBio HiFi reads and genome assembly described in this work are available at NCBI under the Bioproject Number PRJNA987151 and BioSample accession SAMN35889979. The Whole Genome Shotgun project has been deposited at DDBJ/ENA/GenBank under the accession JAUDJH000000000. The version described in this paper is version JAUDJH010000000. The BlobTools contamination check output and BRAKER2 annotation are available on DRYAD (https://datadryad.org/stash/share/AbzGDW3KQ56UTEQNY_tBqhYyC8HVF5-UvetbG028MDU). The code used in this study is available at https://github.com/Chelskoj/Aio/blob/main/Automeris_assembly_annotation Supplemental material available at G3 online.
