# Correction to: Long read genome assembly of *Automeris io* (*Lepidoptera: Saturniidae*) an emerging model for the evolution of deimatic displays

**DOI:** 10.1093/g3journal/jkae048

**Published:** 2024-03-21

This is a correction to: Chelsea Skojec, R Keating Godfrey, Akito Y Kawahara, Long read genome assembly of *Automeris io* (*Lepidoptera: Saturniidae*) an emerging model for the evolution of deimatic displays, *G3 Genes|Genomes|Genetics*, Volume 14, Issue 3, March 2024, jkad292, https://doi.org/10.1093/g3journal/jkad292

In the original publication of this article, there was a numerical error in the beginning of the first sentence of the **Abstract.** This should read: “*Automeris* moths are a morphologically diverse group with 145 described species[…]” instead of: “*Automeris* moths are a morphologically diverse group with 135 described species[…]”.

The emendation has been made to the article.

