# Supplementary material for: Long read genome assembly of Automeris io (Lepidoptera: Saturniidae) an emerging model for the evolution of deimatic displays
Source: G3 (Bethesda). 2024 Feb 7;14(3):jkad292. doi: 10.1093/g3journal/jkad292 (PMC10917498; doi:10.1093/g3journal/jkad292)
Supplement: jkad292_Supplementary_Data [file jkad292_supplementary_data.pdf]

Long read genome assembly of *Automeris io (Lepidoptera: Saturniidae)* an emerging model for the evolution of deimatic displays

Authors Chelsea Skojec[1,2], R. Keating Godfrey[1], Akito Y. Kawahara[1,2]

Affiliations

[1]McGuire Center for Lepidoptera and Biodiversity, Florida Museum of Natural History, University of Florida, Gainesville, FL, 32611 USA

[2]Department of Biology, University of Florida, Gainesville, FL, 32611 USA

Supplemental material

Methods

DNA Isolation

The Quiagen DNeasy Blood & Tissue DNA isolation protocol was modified slightly to improve recovery of high molecular weight fragments. First, to prevent scales and hair from clogging the spin column, samples were lightly scraped and, once homogenized and digested, the tissue was spun down and the upper fraction was used for subsequent DNA isolation steps. Additionally, tubes were not vortexed but only inverted to mix the homogenate buffer. Wide bore pipette tips are also advised to prevent shearing, but were not available for this isolation and may have resulted in smaller fragments.

Supplemental Tables

Table S1. Species used to assess orthologous gene groups in OrthoFinder

Common Name	Species	Peptides annotated	Peptides in analysis	Single-copy	Duplicated	Fragmented	Missing	Complete	Assembly	Annotation Pipeline	Protein FASTA source
Io moth	*Automeris io*	17560	17209	4742	313	53	178	5055	Current work	BRAKER2	Current work
Domestic silkworm	*Bombyx mori*	27309	13881	5175	53	11	47	5228	GCA_014905235.2	NCBI RefSeq	NCBI
Tobacco hornworm	*Manduca sexta*	24854	17354	4837	396	11	42	5223	GCA_014839805.1	NCBI RefSeq	NCBI
Small elephant hawk moth	*Deilephila porcellus*	18356	18147	4783	170	125	208	4953	GCA_905220455.2	BRAKER2	Darwin Tree of Life
Poplar hawkmoth	*Laothoe populi*	17118	16940	4803	161	121	201	4964	GCA_905220505.1	BRAKER2	Darwin Tree of Life
Lime hawk moth	*Mimas tiliae*	16720	16541	4827	174	128	157	5001	GCA_905332985.1	BRAKER2	Darwin Tree of Life
Coxcomb prominent	*Ptilodon capucinus*	17172	16968	4682	205	154	245	4887	GCA_914767695.1	BRAKER2	Darwin Tree of Life
Peacock moth	*Macaria notata*	18126	17964	4719	125	176	266	4844	GCA_927399415.1	BRAKER2	Darwin Tree of Life
Arran brown	*Erebia ligea*	18419	8233	4760	177	150	199	4937	GCA_917051295.2	BRAKER2	Darwin Tree of Life
Silver-spotted skipper	*Hesperia comma*	18967	18725	4615	196	180	295	4811	GCA_905404135.1	BRAKER2	Darwin Tree of Life
Vinegar fly	*Drosophila melanogaster*	30719	13968	*NA*	*NA*	*NA*	*NA*	*NA*	GCA_000001215.4	NCBI RefSeq	NCBI

Cardinal beetle	*Pyrochroa serraticornis*	17588	17216	*NA*	*NA*	*NA*	*NA*	*NA*	GCA_905333025.2	BRAKER2	Darwin Tree of Life

Table S2. Orthogroups corresponding to genes of interest to deimatism

Functional grouping	Orthogroup	Protein name	Gene symbol	Flybase ID
Structural constituents of muscle	OG0000110	Actin gene family	*Actin*	FBgn0286832
	OG0001691	Projectin (bent)	*bt*	FBgn0005666
	OG0007484	Flightin	*fln*	FBgn0005633
	OG0007443	Salimus (kettin)	*sls*	FBgn0086906
	OG0004116	Muscle LIM protein at 84B	*Mlp84B*	FBgn0014863
	OG0003510	Myosin heavy chain	*Mhc*	FBgn0264695
	OG0005895	Myosin light chain	*Mlc2*	FBgn0002773
	OG0007442	myomesin & myosin binding protein	*MnM*	FBgn0035410
	OG0006432	Sarcoglycan δ	*Scgδ*	FBgn0025391
	OG0003704	Syntrophin-like 2	*Syn2*	FBgn0034135
	OG0006064	Tenectin	*tnc*	FBgn0039257
	OG0004816	Tropomyosin 2	*Tm2*	FBgn0004117
	OG0000485	Troponin C (IIIa-like) (*B.mori*)		LOC778498*
	OG0006901	Troponin C 25D (II)	*TpnC25D*	FBgn0031692
	OG0002712	Troponin C 47D (Ib)	*TpnC47D*	FBgn0010423
	OG0002712	Troponin C 73F (Ia)	*TpnC73F*	FBgn0010424
	OG0000485	Troponin C 41C (IIIb)	*TpnC41C*	FBgn0013348
	OG0000485	Troponin C isoform 4		FBgn0033027
	OG0004160	Troponin T (upheld)	*up*	FBgn0004169
	OG0001712	wings up A, Troponin I	*wupA*, *TpnI*	FBgn0283471
Pigmentation	OG0000533	N-acetyl transferase (*B. mori*)	*AANAT*	LOC780849*
	OG0002889	black	*b*	FBgn0000153
	OG0003905	Dopamine transporter	*DAT*	FBgn0034136
	OG0000351	Dopa decarboxylase	*Dcd*	FBgn0000422
	OG0003828	ebony	*e*	FBgn0000527
	OG0007702	punch, GTP cyclohydrolase	*Pu*	FBgn0003162
	OG0006029	straw, Laccase2	*stw*	FBgn0286203
	OG0006725	Optix	*Optix*	FBgn0025360
	OG0004024	tan	*t*	FBgn0086367
	OG0003289	Tyramine β hydroxylase	*Tbh*	FBgn0010329
	OG0005235	yellow-f	*yellow-f*	FBgn0041710

Eye spot formation	OG0002142	Antennapedia	*Antp*	FBgn0260642
	OG0003100	Distal-less	*Dll*	FBgn0000157
	OG0000358	Ultrabithorax (*D. melanogaster*)	*Ubx*	FBgn0003944
	OG0008706	Ultrabithorax (*B. mori*)	*Ubx*	LOC100135425*
	OG0001617	Spalt major	*salm*	FBgn0261648
	OG0006043	Ecdysone receptor	*EcR*	FBgn0000546

* Indicates NCBI gene ID for *Bombyx mori*

Table S3. Repeat content in the *Automeris io* assembly

Element type	Number of elements	Percent of assembly	Length (bp)
Total	1343846	50.36%	246822311
SINEs	15283	0.67%	3274023
LINEs	384732	15.84%	77647128
LTR	60908	4.48%	21948284
DNA	117080	6.90%	33839774
Unclassified	653314	20.81%	101993081
Low complexity	11931	0.12%	564771
Small RNA	203	0.03%	128843
Satellites	13490	0.40%	1970277
Simple repeats	86905	1.11%	5456130

Supplemental Figures

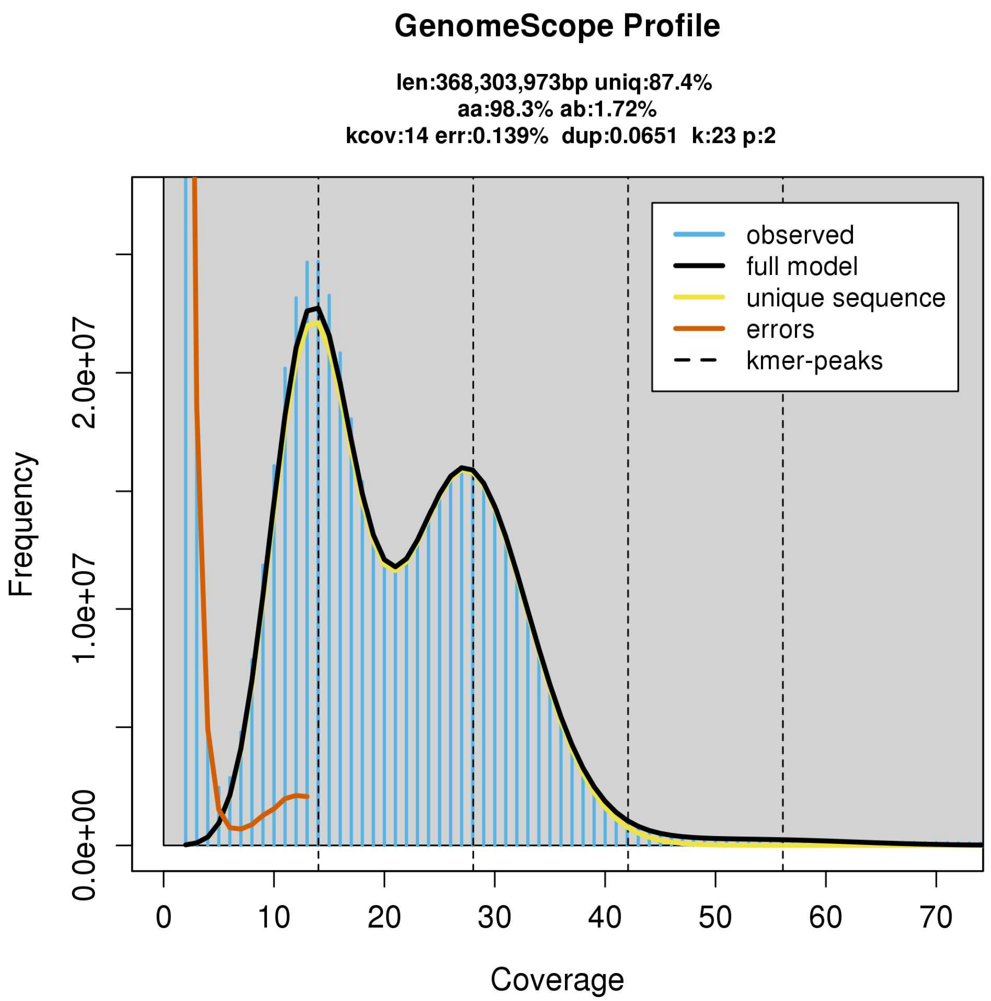

Figure S1. K-mer plot showing homozygous and heterozygous coverage. This plot can be viewed at http://genomescope.org/genomescope2.0/analysis.php?code=vFjBcvan3rVCvp47HGXR

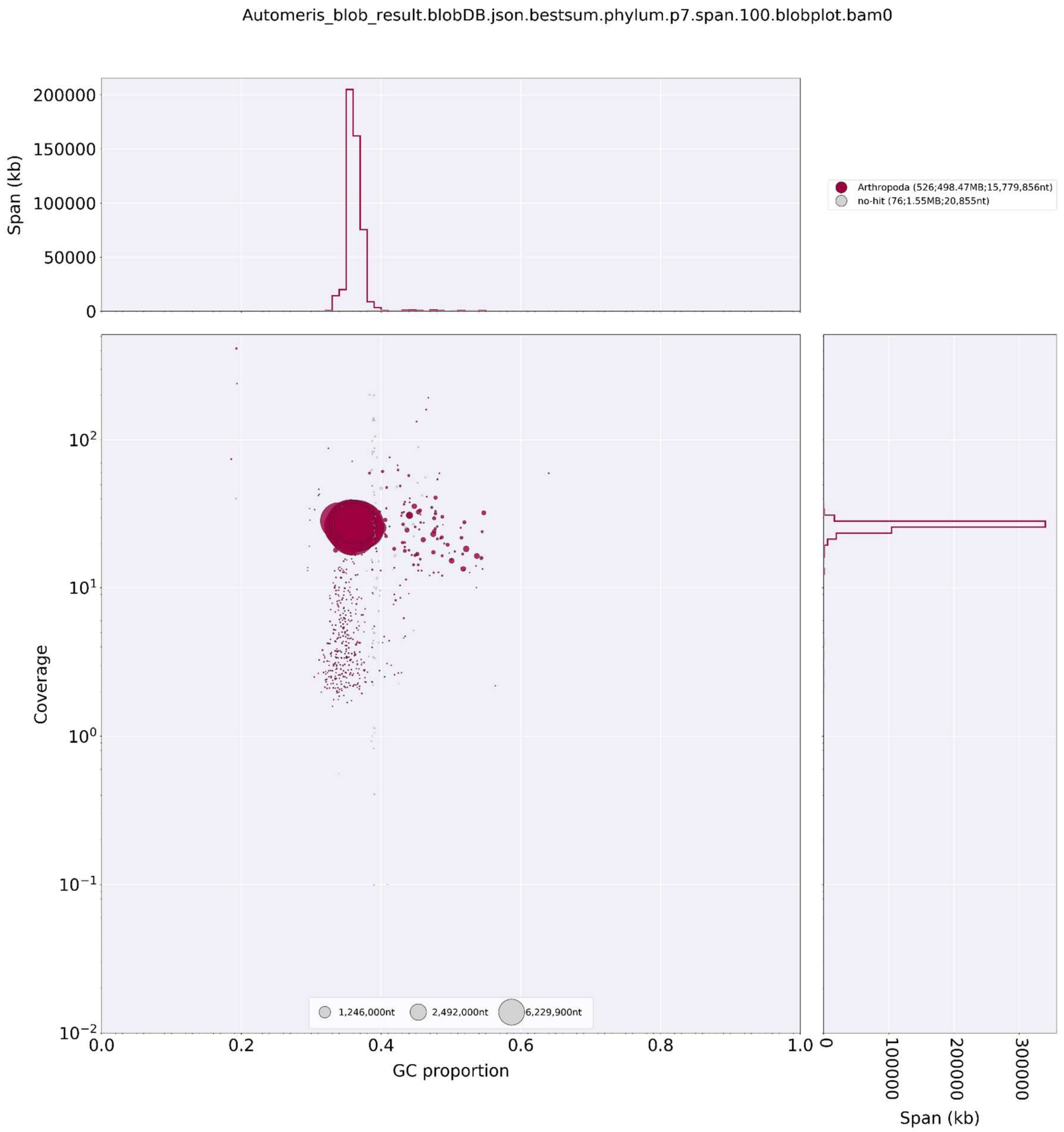

Figure S2. Blobplot showing coverage and GC proportion for *Automeris io* genomic sequences. Circle diameters proportional to sequence length and colored by taxonomic affiliation.

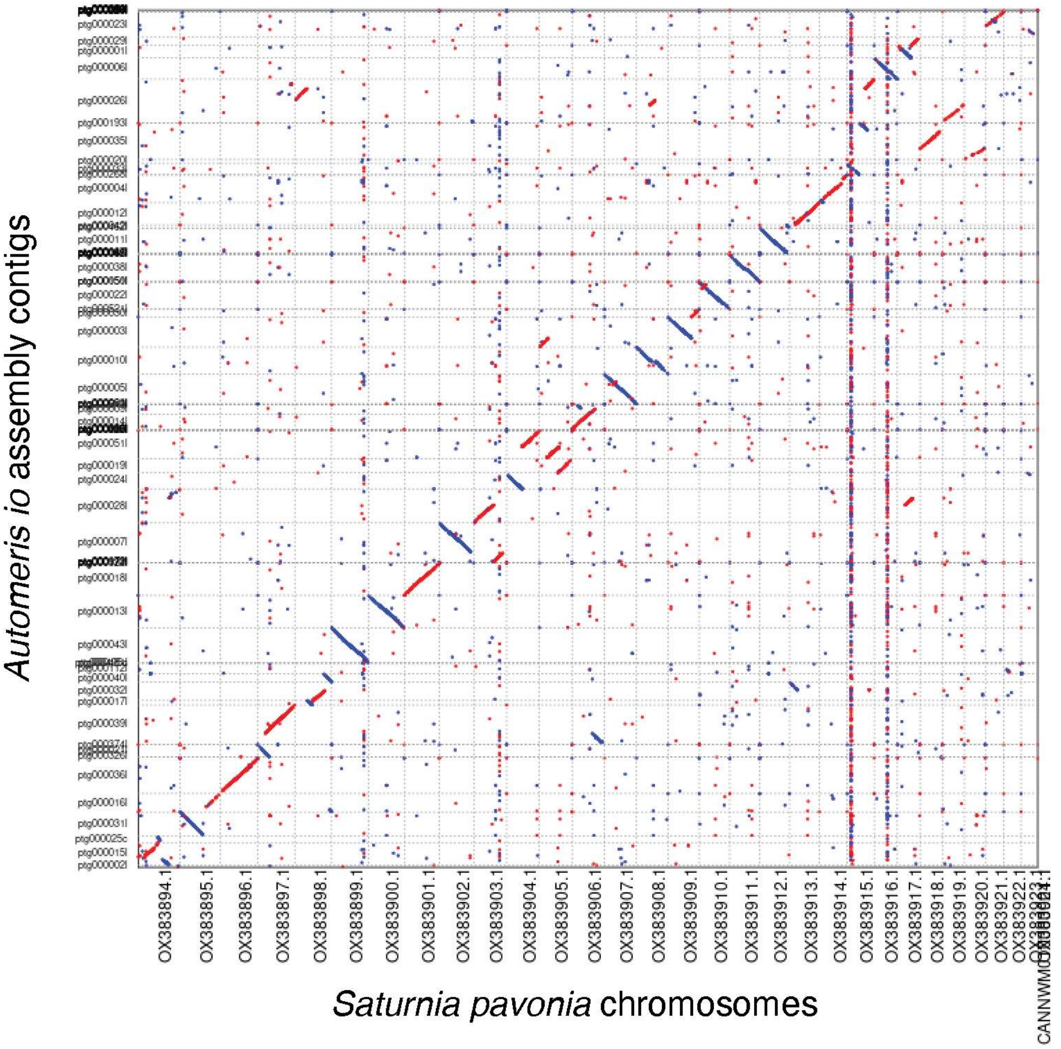

Figure S3. Dot plot showing synteny between the Io moth (*Automeris io*) and the small emperor moth (*Saturnia pavonia*). Each dot represents the position of alignment as determined by nucmer. The colors represent the orientation of the individual alignments, with red indicating consistent orientation and blue indicating inconsistent orientation.
